# Bioinformatic Analysis of *Ixodes ricinus* Long Non-Coding RNAs Predicts Their Binding Ability of Host miRNAs

**DOI:** 10.3390/ijms23179761

**Published:** 2022-08-28

**Authors:** José María Medina, Muhammad Nadeem Abbas, Chaima Bensaoud, Michael Hackenberg, Michail Kotsyfakis

**Affiliations:** 1 Departamentode Genética, Facultad de Ciencias, Universidad de Granada, Campus de Fuentenueva s/n, 18071 Granada, Spain; 2 Laboratorio de Bioinformática, Centro de Investigación Biomédica, PTS, Instituto de Biotecnología, Avda. del Conocimiento s/n, 18016 Granada, Spain; 3 State Key Laboratory of Silkworm Genome Biology, Key Laboratory of Sericultural Biology and Genetic Breeding, Ministry of Agriculture, Southwest University, Chongqing 400715, China; 4 Institute of Parasitology, Biology Centre, Czech Academy of Sciences, 37005 Ceske Budejovice, Czech Republic

**Keywords:** *Ixodes ricinus*, ectoparasite-host interactions, host immunity, RNA-sequencing, lncRNA

## Abstract

*Ixodes ricinus* ticks are distributed across Europe and are a vector of tick-borne diseases. Although *I. ricinus* transcriptome studies have focused exclusively on protein coding genes, the last decade witnessed a strong increase in long non-coding RNA (lncRNA) research and characterization. Here, we report for the first time an exhaustive analysis of these non-coding molecules in *I. ricinus* based on 131 RNA-seq datasets from three different BioProjects. Using this data, we obtained a consensus set of lncRNAs and showed that lncRNA expression is stable among different studies. While the length distribution of lncRNAs from the individual data sets is biased toward short length values, implying the existence of technical artefacts, the consensus lncRNAs show a more homogeneous distribution emphasizing the importance to incorporate data from different sources to generate a solid reference set of lncRNAs. KEGG enrichment analysis of host miRNAs putatively targeting lncRNAs upregulated upon feeding showed that these miRNAs are involved in several relevant functions for the tick-host interaction. The possibility that at least some tick lncRNAs act as host miRNA sponges was further explored by identifying lncRNAs with many target regions for a given host miRNA or sets of host miRNAs that consistently target lncRNAs together. Overall, our findings suggest that lncRNAs that may act as sponges have diverse biological roles related to the tick–host interaction in different tissues.

## 1. Introduction

Ticks are an economically and medically important group of arthropods that feed on the blood of a wide range of vertebrate hosts. The attachment and insertion of tick mouthparts into the host activate the hemostatic processes of blood coagulation, vasoconstriction, and platelet aggregation to reduce blood loss [1,2]. Host innate immunity is induced in response to extended tick feeding and/or long-term tick exposure, and then adaptive immunity is triggered to further improve host defenses [3]. Ticks have adapted to regulate host immune reactions and hemostasis by secreting a complex cocktail of pharmaco-active substances into the host through their saliva [3,4]. There has been—and continues to be—extensive research to identify and functionally characterize the salivary gland or saliva components in ticks. For example, recent studies have tried to identify protective antigens from ticks through the transcriptomes and proteomes of the tick midgut and salivary glands, and the resulting sialomes and mialomes have been annotated and examined for the selection and characterization of antigenic candidates [5,6].

Recent advances in high-throughput sequencing technologies have provided a deeper understanding of non-coding (nc)RNAs—which range from micro (mi)RNAs to long non-coding (lnc)RNAs—in tick tissues [7]. LncRNAs are RNA strands longer than 200 nucleotides that are structurally identical to mRNAs but are not translated into proteins [8]. LncRNAs can regulate coding genes in various ways. For instance, they can modify gene expression through transcription factor activation or repression by binding and localization, chromatin remodeling, imprinting, and enhancer regulation [8]. They are involved in a number of regulatory processes, including post-transcriptional modulation, mRNA processing, and protein trafficking [8]. Chromatin remodeling is modified by lncRNA-induced histone methylation, which alters chromatin structure to increase or decrease DNA access to the transcriptional machinery [9]. LncRNAs participate in this critical role by acting as a protein scaffold with many binding domains that allow methylation and demethylation and interaction with the target histone [9,10]. LncRNAs can also influence translation by binding to mRNAs, increasing or decreasing translation, or triggering mRNA degradation. Furthermore, cytoplasmic lncRNAs can function as miRNA precursors or as “miRNA sponges”: as a result of the competition between lncRNAs and mRNA for miRNA recognition elements, miRNA functions are lost [11]. In eukaryote pathophysiology, these miRNA sponges are widespread regulators of miRNA activity. They are also considered crucial regulators in ectoparasite-host crosstalk, such as during vector–host interactions [12].

LncRNAs have been studied most in humans and animals that are used in medical research [13]. However, there is very little research on lncRNAs in host–parasite interactions in arthropods. In the context of vector-host-pathogen interactions, recent reviews have highlighted a role for small ncRNAs in trans-kingdom and inter-species communication [14,15]. So far, lncRNAs in arthropods have received little attention, and many of their functions are unclear. They could be expressed by the vector to counteract host defenses. It has been proposed that vector lncRNAs are transported in salivary exosomes to the host, where they act as host miRNA sponges to disrupt natural defense reactions; however, this hypothesis still requires validation [12].

The main objective of the present study was to elucidate the biological roles of lncRNAs in ectoparasite–host interactions, with bioinformatic means and through a consensus strategy, and particularly their potential role in tick–host interactions. Thus, we aimed to offer a new mechanistic understanding to inform the development of novel treatments for various diseases. *Ixodes ricinus* was used as a model ectoparasite, as it is a common vector of human and animal diseases, particularly in Europe.

## 2. Results

### 2.1. Summary of Transcriptome Assembly and lncRNA Isolation

By means of transcriptome assembly and lncRNA isolation, a total of 14,079, 677,278, and 358,278 lncRNAs were isolated from the transcriptome from midguts and salivary glands (MG-SG lncRNAs), salivary glands only (SG lncRNAs), and whole body samples (WB lncRNAs), respectively. The relatively low number of lncRNAs obtained from the transcriptome from midguts and salivary glands reflects the additional and more stringent steps taken in its transcriptome assembly process as explained in the Material and methods correspondent section.

To generate a consensus set we clustered the three sets of lncRNAs. The consensus lncRNA was defined as the longest transcript from a given cluster that could be detected in the MG-SG dataset (the most reliable lncRNA set, since the selection was more stringent) and at least in one of the other two transcriptomes. In this way we obtained a final set of 3118 consensus lncRNAs. 

With respect to coding RNAs, we isolated 12,158, 55,825 and 47,472 coding sequences from the transcriptome from midguts and salivary glands (MG-SG coding RNAs), salivary glands only (SG coding RNAs), and whole body samples (WB coding RNAs), respectively. We followed the same protocol that we used for lncRNAs to get a final consensus set of 5659 coding RNAs. 

### 2.2. Expression Patterns of lncRNAs in the Midgut and Salivary Glands under Different Feeding Treatments

**Figure 1:**
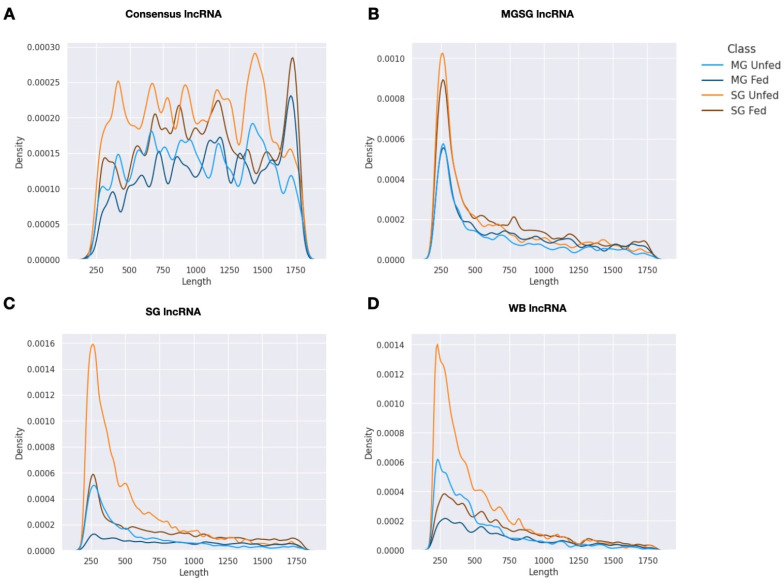
Length distribution of differentially expressed lncRNAs in the midgut and salivary glands of unfed and fed *Ixodes ricinus*. (**A**) Length distribution of significantly differentially expressed consensus lncRNAs (**A**), MG-SG lncRNAs (**B**), SG lncRNAs (**C**), and WB lncRNAs (**D**) upregulated in unfed and fed ticks in the midguts and salivary glands. Different lines represent lncRNAs upregulated or downregulated upon feeding in midgut (MG fed, MG unfed) and salivary glands (SG fed, SG unfed) with respect to midgut and salivary gland dataset (BioProject: PRJNA716261).

Initially, four *I. ricinus* sets of lncRNAs were analyzed: consensus lncRNAs, MG-SG lncRNAs, SG lncRNAs, and WB lncRNAs. To assess the quality of these sets, we evaluated and visualized the length distributions (Figure 1). LncRNAs obtained from the individual projects show a strikingly different distribution compared to the consensus sequences. Short lncRNAs are clearly overrepresented (maximum around 250 nt) in the individual sets while the consensus shows a much more homogeneous distribution. We interpret this finding as the successful recovery of longer sequences by means of the consensus approach i.e., lncRNAs that are represented decently only in one of the individual sets. 

Therefore, we will base this study in the consensus transcriptome. 

**Figure 2:**
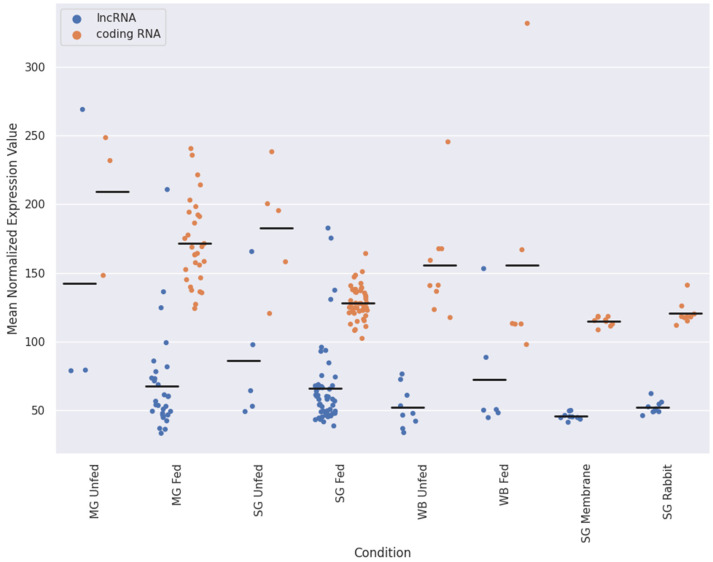
Expression analysis of lncRNAs and coding RNAs for different conditions of the samples retrieved for this study. The expression of lncRNAs and coding RNAs was analyzed for different feeding treatments: MG unfed, MG fed, SG unfed, and SG fed correspond to samples from midgut and salivary glands from ticks not fed or in the slow feeding phase, respectively (BioProject PRJNA716261); WB unfed and WB fed correspond to samples from whole body from ticks not fed or that were in the slow feeding phase, respectively (BioProject: PRJNA395009); lastly, SG Membrane and SG rabbits show the expression value for samples from salivary glands only that were fed on rabbits or from a membrane (BioProject: PRJNA312361). Mean values per condition are marked with a black line.

Since this is, to our best knowledge, the first time that lncRNAs are analyzed in *I. ricinus*, we first characterize the overall expression levels of lncRNAs in comparison to coding sequences. Figure 2 shows the mean normalized expression values for lncRNAs and coding sequences as a function of tissue or feeding state. The mean expression value of the consensus lncRNA was consistent among the different conditions representing approximately 30% of the total expression. We found some samples with strongly increased lncRNA expression, interestingly most of which belong to unfed or early feeding samples. 

**Figure 3:**
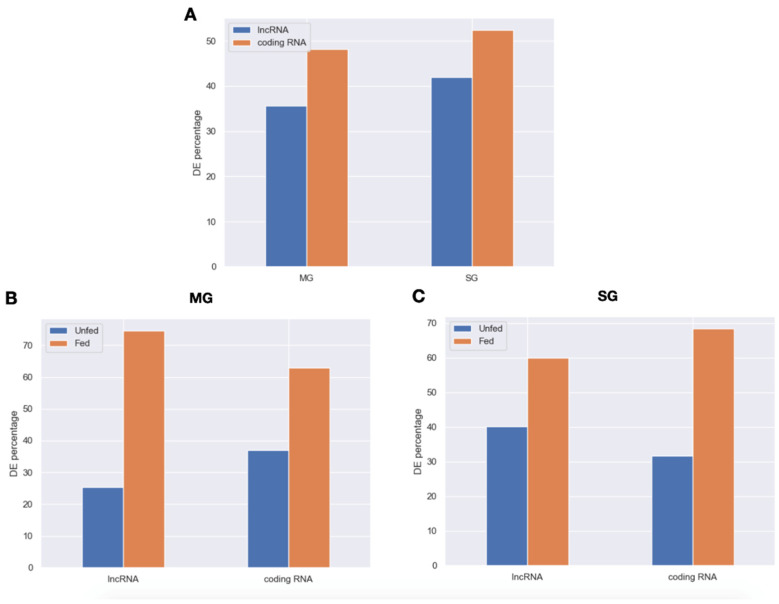
Statistics of differentially expressed lncRNAs and coding RNAs for midgut and salivary glands of *Ixodes ricinus*. (**A**) Percentage of lncRNAs and coding RNAs differentially expressed in midgut and salivary glands. (**B**) Distribution of DE lncRNAs and cRNAs between feeding threads in midgut. (**C**) Distribution of DE lncRNAs and cRNAs between feeding threads in salivary glands.

Additionally, to better understand the impact of lncRNA in ticks that are not feeding from a host and ticks that are actively feeding, we analyzed the differential expression of lncRNAs and coding RNAs between samples from unfed and fed ticks for both midgut and salivary glands (PRJNA716261). Differentially expressed (DE) lncRNAs and coding RNAs in midgut and salivary glands are displayed using volcano plots (Figure S1). For midgut, we found a total of 1110 DE lncRNAs (35.6% of the total of consensus lncRNAs) and 2727 DE coding RNAs (48.2% of the total of consensus coding RNAs). Regarding the salivary glands, 1311 lncRNAs (42% of the consensus lncRNAs) and 2965 coding RNAs (52.4% of the consensus coding RNAs) were differentially expressed between feeding treatments (Figure 3A). The DE lncRNAs and coding RNAs were then classified in two classes: (1) overexpressed when *I. ricinus* is not feeding and (2) overexpressed when *I. ricinus* is actively feeding. Here, we found that, for both tissues, the majority of DE lncRNAs and DE coding RNAs are overexpressed when *I. ricinus* is actively feeding (Figure 3B,C). Specifically, in midgut, 74.6% of the lncRNAs and 63% of coding RNAs were overexpressed in fed ticks whereas 59.9% and 68.4% of the lncRNAs and coding RNAs, respectively, were overexpressed in salivary glands of feeding ticks.

### 2.3. Target Prediction and KEGG Analysis of the Consensus Differentially Expressed lncRNAs

In order to explore putative roles of lncRNAs in tick feeding, we analyzed the putative functions of host miRNA targets. Applying miRNAconsTarget from sRNAtoolbox [16], we carried out a target prediction using human microRNAs from MirGeneDB v2.1 [17] and DE lncRNA sequences. We selected and functionally analyzed those miRNAs with the overall highest ratio of targets. We found that hsa-mir-5683, hsa-mir-29b-3p, and hsa-mir-154-3p are the miRNAs with the highest ratio of targets in feeding regulated lncRNAs. According to MirPath [18], these three miRNAs target genes are involved in focal adhesion and PI3K-Akt signaling pathway. They also target genes with a role in ECM-receptor interaction, fatty acids metabolism and biosynthesis, among other functions. Regarding the salivary glands, the miRNAs with the highest ratio of targets for lncRNAs upregulated in fed ticks were hsa-miR-4664-3p, hsa-miR-431-5p, and has-miR-28-3p. Among the KEGG pathways enriched for the genes they target, we identified Hippo signaling pathway, adherens junction, cell cycle, choline metabolism in cancer, and mucin type O-Glycan biosynthesis. All KEGG pathways enriched for miRNAs in both tissues, *p*-value, number of genes involved and number of miRNAs targeting genes involved in the KEGG pathway may be found in File S1. 

### 2.4. Analysis of the Reproducibility of the Differential Expression of lncRNAs

Reproducibility is a key concept to increase the reliability of scientific results. We took advantage of having two different studies with samples from salivary glands of ticks fed on rabbits till 24 and 72 h obtaining differentially expressed lncRNAs and coding sequences for both studies (PRJNA716261, PRJNA312361). The distribution of differential expression in form of Volcano plots can be found in Figure S2. 

**Figure 4:**
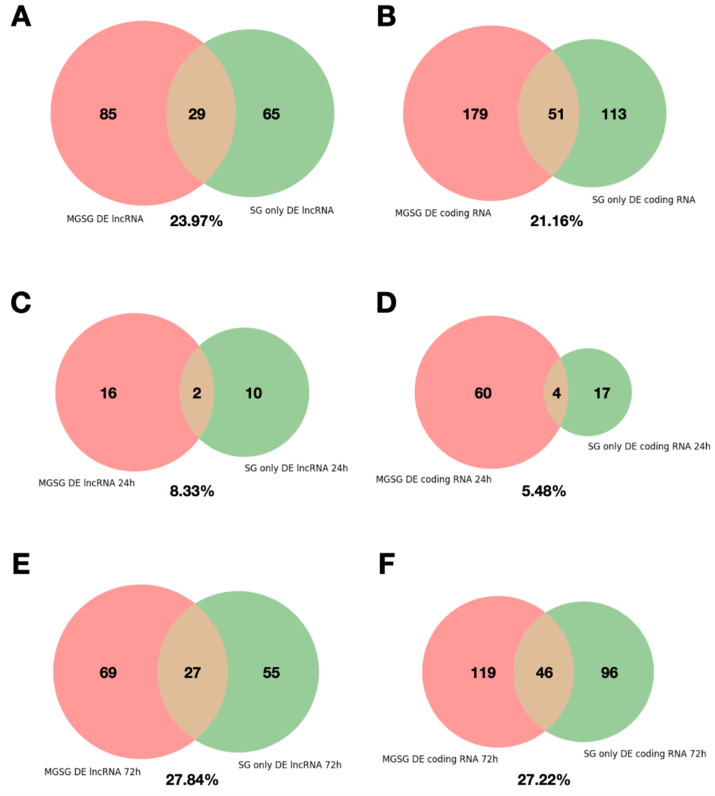
Analysis of the reproducibility of the differential expression of consensus lncRNAs and coding RNAs between the studies MGSG (PRJNA716261) and SG only (PRJNA312361) in salivary glands. The Venn diagrams shows the overlap between (**A**) DE lncRNAs in MGSG samples and DE lncRNAs in SG only samples, (**B**) DE coding RNAs in MGSG samples and DE coding RNAs in SG only samples, (**C**) DE lncRNAs downregulated at 72 h of feeding in MGSG and SG only samples (**D**) DE coding RNAs downregulated at 72 h of feeding in MGSG and SG only samples, (**E**) upregulated lncRNAs at 72 h of feeding in MGSG and SG only samples and (**F**) upregulated coding RNAs at 72 h of feeding in MGSG and SG only.

We show that the percentage of differentially expressed sequences that coincide between the two studies were similar for lncRNAs and coding RNAs (23.97% and 21.16%, Figure 4A,B). Figure 4C–F shows a breakdown of these numbers into up and downregulated at 72 h compared to 24 h. Interestingly, the overlap between both studies is much higher for upregulated sequences (around 27%, Figure 4E,F) than for downregulated ones (8% for lncRNAs, Figure 4C,D).

### 2.5. Prediction of Sponge Candidates and Functional Analysis

Cytoplasmic lncRNAs can serve as “miRNA sponges” competing with mRNA molecules for miRNA binding which can lead to the loss of miRNA function. To explore the possibility that tick lncRNAs play a role in the feeding process we explore two different sponge models: (i) high frequency targets and (ii) recurrent host miRNA combinations. We first performed target prediction analysis for all consensus lncRNAs as mentioned before. The results of target prediction can be found in File S2. As a negative control we carry out target prediction for a randomized set of consensus lncRNAs using shuffleseq (File S3). Target prediction for the consensus and randomized lncRNAs is shown for the top 200 miRNA-lncRNA combinations with the highest number of targets (Files S4 and S5). Most lncRNAs of this set contained more targets than its randomized version for the same miRNA (Files S6 and S7 for normalized target counts). 

Next, we tried to extract the subset of lncRNAs with the highest possibility of being sponge candidates applying three different approaches: (i) highest number of targets for a given miRNA (Files S4 and S5), (ii) highest density of targets (Files S6 and S7), (iii) combination of microRNA target sites. For all, a negative control was performed using shuffled sequences. Significant microRNA combinations were detected using the Apriori algorithm. Thus, we identified sets of miRNAs that consistently target lncRNAs (File S8). To further understand the function of lncRNAs that may act as sponges, we selected the best sponge candidates from each analysis defined as the consensus lncRNA with the greatest number of targets for a miRNA, the consensus lncRNAs with the greatest number of targets per kb for a given miRNA, and three groups of consensus lncRNAs, with no common lncRNAs between them, that are significantly and consistently targeted by a group of host miRNAs.

**Figure 5:**
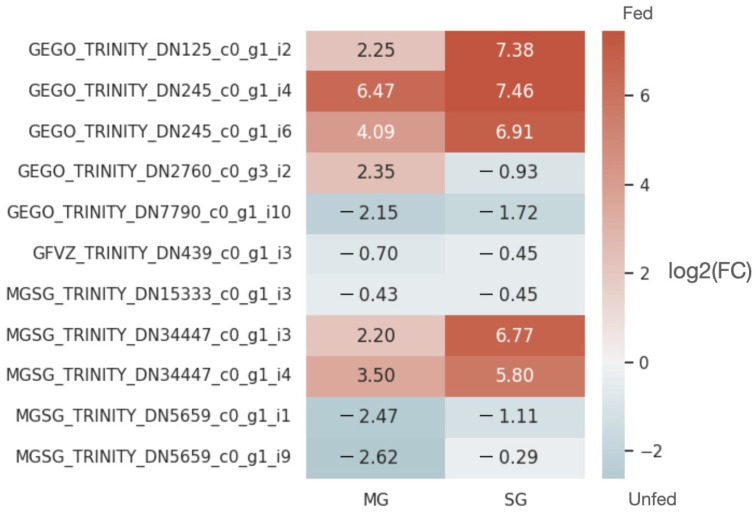
Differential expression of the sponge candidates in the midgut and salivary glands of unfed and fed *Ixodes ricinus*. The heatmap displays the logarithm fold change to base 2 between the unfed and fed *I. ricinus* for each sponge candidate in both the midgut and salivary glands. A logarithm of fold change to base 2 greater than 0 indicates that the sponge candidate is upregulated in fed ticks, while a logarithm of fold change to base 2 less than 0 indicates that the sponge candidate is upregulated in unfed ticks.

A heat map was constructed for potential sponge candidates to analyze variability in lncRNA expression in unfed and fed midguts and salivary glands. The expression of many DE lncRNAs was strongly increased by feeding in the salivary glands compared with the midgut (Figure 5). To further understand the function of the sponge candidates, we used miTALOS v2.0 [19] to characterize human miRNAs or miRNA combinations which may be target by tick lncRNAs. These individual host miRNAs or miRNA combinations were found to control hemostasis-associated (e.g., glycosaminoglycan biosynthesis–heparan sulfate/heparin) or immune response (e.g., TGF-β signaling pathway, BMP signaling, TCF-dependent signaling in response to WNT, and PDGF pathway)-associated pathways in humans. In addition, miRNAs or miRNA combinations also had targets on a variable number of genes in their corresponding pathways (File S9), suggesting potential functions of lncRNAs in tick feeding and ectoparasite–host interactions.

## 3. Discussion

The molecular mechanisms of action and physiological impacts of lncRNAs are an intense focus of applied human and animal research [13]. However, there is currently very little literature on the identification and functional analysis of lncRNAs in tick–host interactions, limiting our understanding of lncRNAs and their involvement in ectoparasite–host interactions. Therefore, to evaluate the potential regulatory role of lncRNAs in *I. ricinus*–host interactions, we investigated the differential expression of lncRNAs in the midguts and salivary glands of unfed and fed *I. ricinus* ticks. Previous studies have shown that lncRNAs are involved in different physiological processes such as mRNA processing, post-transcriptional regulation, and protein trafficking [8,9]. In addition, cytoplasmic lncRNAs can also act as miRNA precursors or as “miRNA sponges”, where they compete with mRNAs for miRNA recognition elements, causing the loss of miRNA function [11]. For this reason, we performed transcriptome assembly, lncRNA identification, differential expression analysis, and functional in silico analysis to identify lncRNAs in the midguts and salivary glands of *I. ricinus* ticks under different feeding treatments (unfed and fed) with potential functions related to tick-host interactions. We focused on midgut and salivary gland-related differentially expressed lncRNAs and showed that the production of lncRNAs in the salivary gland may play a role in the ectoparasite–host interaction.

*Ixodes ricinus* and other Ixodid ticks are unique in their long period of attachment to the host [20,21]). The tick salivary gland mediates various activities that ensure the tick’s biological success during feeding. The tick salivary gland produces biologically active molecules that facilitate blood meal acquisition [4]. Therefore, the composition of tick saliva requires careful and comprehensive molecular resolution to understand the complex feeding biology of ticks. Such resolution could underpin the discovery of pharmacologically active molecules of clinical interest. So far, many transcript and protein profiling studies of tick saliva have been conducted at different developmental stages, sexes, and feeding conditions in different tick species [22,23,24]. While many studies have attempted to determine the complex nature of saliva or the salivary gland, this is the first study to investigate ncRNAs in *I. ricinus*. We identified many potential lncRNAs from *I. ricinus* and ensured that these potential lncRNAs had the most reliability and reproducibility we could afford by analyzing different transcriptome projects. Initial analysis about lncRNAs expression showed that the expression level of lncRNAs compared to coding RNAs is stable among studies. Additionally, a proof about the reproducibility of differential expression between studies showed that the reproducibility of lncRNAs was similar to the reproducibility of coding RNAs. Next, we analyze the differential expression of the consensus lncRNAs for different feeding treatments in midgut and salivary glands and compared them with the differential expression calculated for a consensus set of coding RNAs. The results obtained showed that even though the percentage of consensus lncRNAs being DE is lower than the one for coding RNAs in both midgut and salivary glands, most of the DE lncRNAs were upregulated in fed ticks, especially in midgut. This suggests that the predicted consensus lncRNAs have a role in the feeding of *I. ricinus* in both midgut and salivary glands. This idea is supported by the KEGG enrichment analysis of miRNAs that target lncRNAs upregulated in fed ticks. This analysis showed that lncRNAs may disrupt the function of host miRNAs that may be harmful for the tick or beneficial for the host immune response. Specifically, the three miRNAs with the highest ratio of targets for lncRNAs upregulated in fed ticks were involved in processes as focal adhesion and PI3K-Akt signaling pathway. Focal adhesion has been related to epithelial motility and mucosal healing of gut due to its involvement in cell motility, proliferation, and survival [25]. Additionally, PI3K-Akt signaling pathway has been reported to control the epithelial proliferation of the gut [26]. Although further research is needed in this field, we found that lncRNAs in midgut are targeted by miRNAs involved in crucial functions for the healing and correct operation of the midgut. Regarding the salivary glands, we identified interesting KEGG pathways as Hippo signaling pathway, which has been reported to maintain the immune homeostasis in immune cells [27]; adherens junction, which is involved in the innate immune response to endotoxins [28]; and biosynthesis of mucin type O-Glycans, which are involved in the inflammatory response [29]. Here, we found that these miRNAs are involved in the immune response of the host, so disrupting its function may be beneficial for the tick interaction with the vertebrate host. All what was discussed here may establish future lines of research in the field of tick–host interactions and its treatment and prevention.

Target and sponge candidate prediction analysis showed that the salivary glands contain a large variety of lncRNAs that may function in controlling host homeostasis and immune responses, most likely by sponging host miRNAs. Ahmad et al. [12] hypothesized that vector lncRNAs are transported to the host in salivary exosomes, where they act as “sponges” of host miRNAs to disrupt natural defense reactions; however, this hypothesis still requires experimental validation. Furthermore, we analyzed human miRNAs or miRNA combinations to understand tick lncRNA functions using a bioinformatics tool. In doing so, we found that miRNA or miRNA combinations likely to be sponged by tick lncRNAs regulate a variety of genes involved in host hemostasis and immune pathways. We observed that the targeted host miRNAs significantly affected the hemostasis-associated pathway, glycosaminoglycan biosynthesis–heparan sulfate/heparin. Heparin is crucial for the catalysis of antithrombin III (a plasma enzyme), enhancing its activity in vertebrates. Antithrombin III inactivates some activated serine proteases of the coagulation cascade, most importantly activated thrombin and factor X [30]. Furthermore, several immune pathways were influenced by the miRNAs, for example, transforming growth factor-beta (TGF-β) signaling, which is a potent cytokine regulator with various effects on hemopoietic cells. The key biological role of TGF-β in the immune system is to maintain tolerance by modulating lymphocyte proliferation, differentiation, and survival [31,32]. WNT is another immune signaling pathway that might be regulated by tick sponge candidates. WNT ligands bind Frizzled and lipoprotein receptor-related protein to induce the canonical WNT signaling pathway, which inactivates the destruction complex, stabilizes, and promotes the nuclear translocation of β-catenin, and subsequently activates T-cell factor/lymphoid enhancing factor (TCF/LEF)-dependent transcription [33,34]. Thus, ticks are likely to “cargo” lncRNAs into the host body via saliva or salivary exosomes, which may then sponge host miRNAs or miRNA combinations important for host hemostasis and the innate and adaptive immune responses, ultimately facilitating tick feeding on the host. 

Here, we analyzed lncRNAs and their expression in fed and unfed pathogen-free *I. ricinus* ticks and showed evidence of lncRNAs having an important role in *I. ricinus*–host interaction. This study leads to new research about lncRNAs in ectoparasites and to the comparison of lncRNA behavior between them. Additionally, differences in the expression of lncRNAs between pathogen-infected and pathogen-free ectoparasites has been previously reported in *Ae. aegypti* and *Ae. albopictus* [35,36]. Therefore, a comparison between lncRNA expression in ticks infected by pathogens and pathogen-free ticks and the integration of this analysis with strategies for prediction of sponge candidates may help the research community to have a deeper understanding in the role of lncRNAs in the tick-host interaction and their pathogenicity. Ultimately, this study, for the first time, provides in silico evidence on the putative role of tick lncRNAs as sponge candidates. Future studies will focus on evaluating the biological functions of these sponge candidates, in particular in the context of the ectoparasite–host interaction. 

## 4. Materials and Methods

### 4.1. Transcriptome Assembly, Expression Analysis and Consensus lncRNA Isolation

This study used RNA-seq data from *Ixodes ricinus* available in NCBI under the BioProject accessions PRJNA716261, PRJNA312361, and PRJNA395009 [37,38,39]. RNA-seq data from BioProject PRJNA716261 consisted of 88 *Ixodes ricinus* samples exposed to different feeding treatments from two different tissues: 55 samples from the salivary glands, and 33 from the midgut. BioProject PRJNA31236 contained RNA-seq data from 18 salivary gland samples from *Ixodes ricinus*, while BioProject PRJNA395009 contained RNA-seq data from 15 whole body samples of *Ixodes ricinus*. All raw RNA-seq reads were quality filtered and trimmed using Trim Galore [40]. The quality of raw and trimmed reads was determined using FastQC [41]. De novo RNA-seq assemblies from the different BioProjects were constructed separately using Trinity v2.8.6 [42], with a maximum memory usage of 300 gigabytes. Since the RNA-seq data from BioProject PRJNA716261 originated from two different tissues, some additional steps were taken to obtain reliable transcriptomes. First, using Trinity, we created de novo assemblies of the midgut and salivary glands separately. These assemblies were condensed and clustered using CD-HIT v4.8.1 [43]. Contigs with at least 98% identity and at least 80% alignment coverage for the shorter sequences were clustered. Since this study focused on analyzing lncRNAs under different feeding treatments, we calculated the contig expression of all the assemblies for the fed and unfed conditions for both midgut and salivary glands using the RNA-seq data from BioProject PRJNA716261. The contig expression was analyzed using RSEM v1.3.3 [44] and Bowtie2 [45]. Since the dataset coming from midguts and salivary glands is the one with the greatest number of samples (88), which were isolated from single individuals which were siblings, thus reducing genetic variation [37], we decided to give it a major role in the consensus strategy and to make additional and more stringent steps to get from it the most reliable set of lncRNAs and coding RNAs that we could afford. Therefore, to remove possible assembly artifacts from the transcriptome assemblies from the midgut and salivary glands, we applied an expression filter to the contigs. The samples in this BioProject were unfed or fed for different time periods during the slow-feeding phase of the tick. To remain in further analyses, a contig from the transcriptome assembly from the midgut and salivary glands needed to have at least five FPKM in all the samples belonging to the unfed condition or in all the samples that were fed to a given time point. 

For every assembled transcriptome, contigs with open reading frame (ORFs) longer than 50 amino acids were isolated using TransDecoder v5.5.0. The ORFs were then aligned to the Swiss-Prot [46] protein database using BlastP v2.10.1+ [47], with an E-value cut-off of 10^−5^. Contigs with ORFs aligning with the protein database were considered coding RNAs.

To obtain lncRNAs, ncRNAs between 200 bp and 1800 bp were isolated. To avoid possible assembly artifacts and to obtain reliable predicted lncRNAs, consensus lncRNAs were obtained. Using CD-HIT [43], we clustered three sets of lncRNAs: (1) lncRNAs obtained from the midgut and salivary gland (MG-SG lncRNAs); (2) lncRNAs obtained from the salivary gland (SG lncRNAs), and (3) lncRNAs obtained from whole tick bodies (WB lncRNAs). Finally, we retrieved the best representative for each cluster with at least one lncRNA from MG-SG lncRNAs, as this was the most reliable lncRNA set through the application of a stringent expression filter, and at least one lncRNA from the salivary gland or whole body datasets. Thus, we obtained a final set of consensus lncRNAs. For purposes of comparison, we also obtained a consensus set of coding RNAs by following the steps mentioned before. All lncRNAs and cRNAs identified in this study can be downloaded from the Downloads section of IxoriDB [48]. 

### 4.2. Differential Expression Analysis

Infecting and feeding on a host are crucial tick activities during which the tick needs to avoid the host immune system [49]. We hypothesized that ticks produce lncRNAs that act as sponges, trapping miRNAs associated with the host immune system or other processes harmful to the tick. To understand this phenomenon, we analyzed the differential expression (DE) of lncRNAs in the unfed and fed states.

To obtain DE under different feeding conditions, we normalized the expected counts provided by RSEM using the TMM method [50]. Then, DE analysis was executed using EdgeR v3.36.0 [51]. Basic statistics and length distribution analysis were conducted for DE lncRNAs and DE coding RNAs using a homemade Python script.

### 4.3. Target Prediction

The set of mature *H. sapiens* miRNAs was obtained using MirGeneDB v2.1 [17]. Target prediction for the consensus differentially expressed lncRNAs and mature miRNAs from *H. sapiens* was carried out using miRNAconsTarget from sRNAtoolbox [16]. Specifically, the SEED, MIRANDA, and TS (TargetSpy) algorithms were used, and only those differentially expressed lncRNAs confirmed to have targets for miRNAs were retained for further analysis.

### 4.4. Functional In Silico Analysis of Differentially Expressed lncRNAs

To determine the functional variation between consensus differentially expressed lncRNAs in the unfed and fed conditions, we performed functional in silico analysis based on the miRNAs targeting consensus differentially expressed lncRNAs. For this purpose, we retrieved the set of mature miRNAs from *Homo sapiens* using MirGeneDB v2.1 [17]. Target prediction provided us with the number of targets that a miRNA has for the different sets of DE lncRNAs. We normalized the number of targets in each set using the miRNA with the maximum number of targets in each set. We then calculated the ratio of normalized number of targets for lncRNAs upregulated in fed ticks by dividing it by the number of targets for lncRNAs upregulated in unfed ticks for both midgut and salivary glands. We selected three miRNAs for each tissue with the highest ratio and used them to perform KEGG pathway enrichment analysis using the information provided in DIANA-miRPath v3.0 [18]. 

### 4.5. Sponge Candidates and Functional In Silico Analysis

The final objective of this study was to predict lncRNAs from the consensus set of lncRNAs with the highest potential to be sponge candidates. We applied three different approaches to find the best sponge candidates. The first was based on determining which lncRNAs had the highest number of miRNA targets. The second aimed to maximize the number of targets per kb for a miRNA in a lncRNA and was used to normalize the number of targets with the length of the lncRNA. Finally, rather than focusing on lncRNAs with a high number of targets for a single miRNA, we focused on lncRNAs with multiple miRNAs targets. We used the Apriori algorithm to analyze this phenomenon. This algorithm identifies combinations of miRNAs that together target a set of lncRNAs in a statistically significant way. Thus, we obtained sets of miRNAs that consistently targeted lncRNAs together. Lastly, we took as the best sponge candidates the consensus lncRNAs with the greatest number of targets for a miRNA, the greatest number of targets per kb for a given miRNA, and three groups of consensus lncRNAs with no common lncRNAs between them significantly and consistently targeted by a group of miRNAs.

As described above, our functional in silico analysis of lncRNAs is based on the miRNAs that target them. We assigned a function to the miRNA and sponge candidate combinations using miTALOS v2.0 [19]. Using this web tool, we acquired information about the pathways in which the miRNAs participate and the location of the genes they target in those pathways. 

## 5. Conclusions

Here, we analyzed and detected lncRNAs through consensus methods from publicly available RNA-seq data from the salivary glands and midguts (BioProject: PRJNA716261), salivary glands alone (BioProject: PRJNA312361), and whole bodies (BioProject: PRJNA395009), showing stable overall expression and reproducible response upon feeding challenges. Overall, our bioinformatics analysis suggests that a subset of lncRNAs might act as host microRNA sponges with specific functions in tick–host interactions. We provide here a consensus set of lncRNAs that opens the door for future functional assays to obtain a deeper understanding of the function of these lncRNAs at the tick-vertebrate host interface, which could offer a new mechanistic understanding and may revolutionize the development of novel treatments for tick-borne diseases.

## Data Availability

The data presented in this study are available in the article and Supplementary Material. The RNA-seq data used in this study is available in NCBI under the BioProject accessions PRJNA716261, PRJNA312361, and PRJNA395009. The lncRNA data isolated in this study is available in the “Downloads” section of IxoriDB [48].

## Supplementary Materials

The following supporting information can be downloaded at: https://www.mdpi.com/article/10.3390/ijms23179761/s1.
