# Supplementary figures and images for: Bioinformatic Analysis of Ixodes ricinus Long Non-Coding RNAs Predicts Their Binding Ability of Host miRNAs
Source: Int J Mol Sci. 2022 Aug 28;23(17):9761. doi: 10.3390/ijms23179761 (PMC9456184; doi:10.3390/ijms23179761)

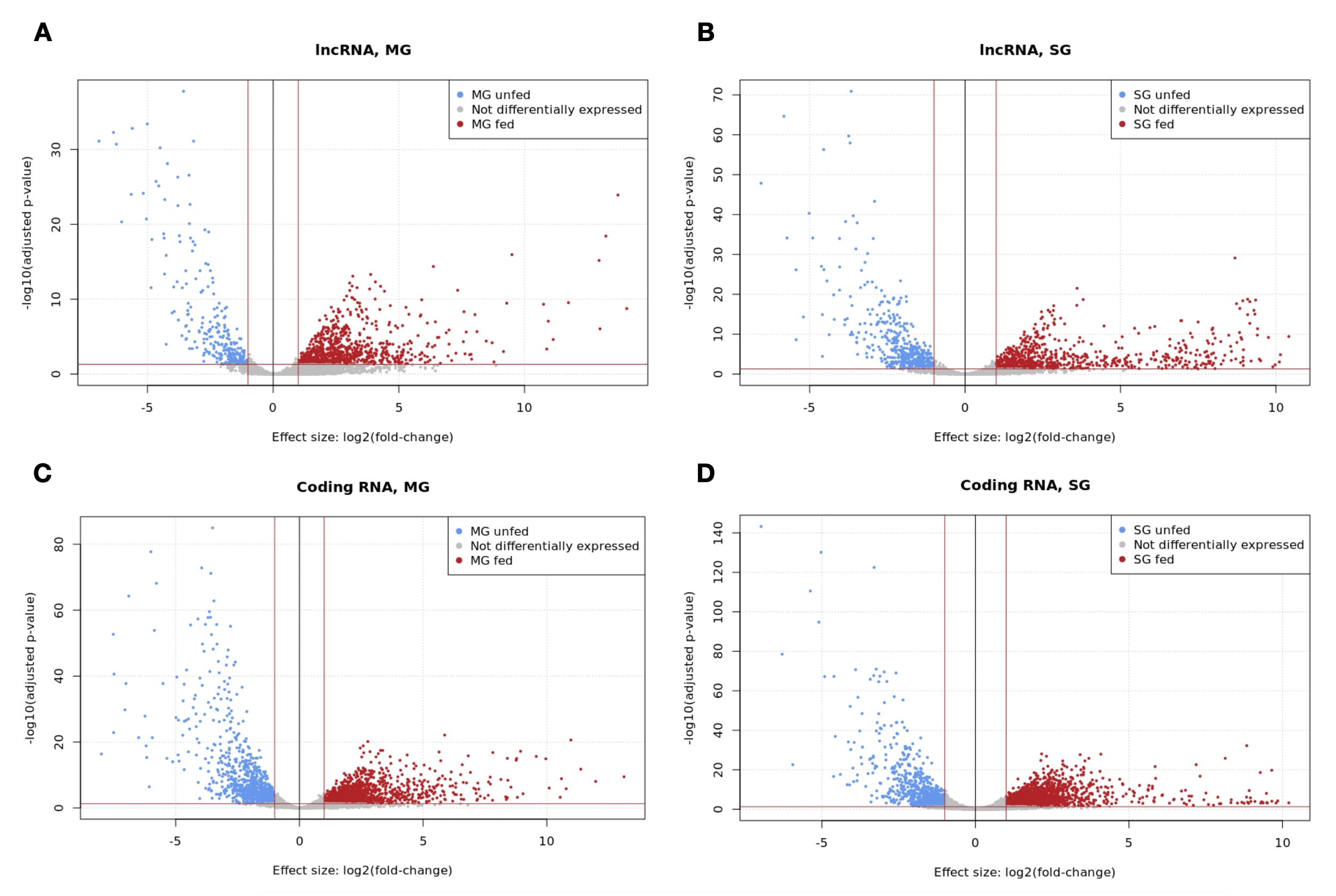

Supplement: Supplementary file 1 [file ijms-23-09761-s001.zip › Figure_S1.jpg]

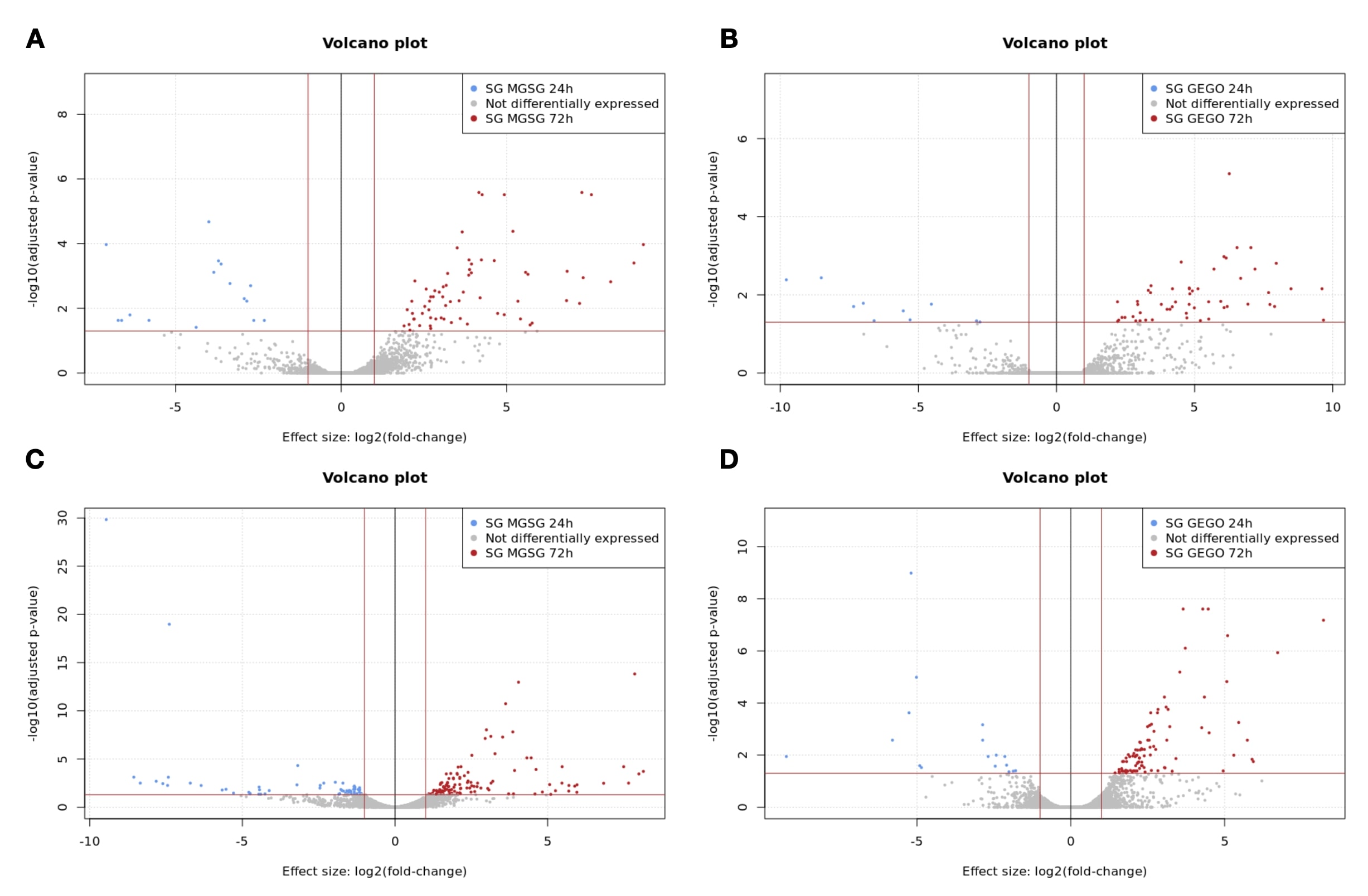

Supplement: Supplementary file 1 [file ijms-23-09761-s001.zip › Figure_S2.jpg]
